# An evaluation of the 2012 measles mass vaccination campaign in Guinea

**DOI:** 10.11604/pamj.2014.17.4.2475

**Published:** 2014-01-08

**Authors:** Jean Gerard Tatou Doumtsop, Emmanuel Roland Malano, Ibrahima Telly Diallo, Camara Sirimah

**Affiliations:** 1 Ministry of Health and Public Hygiene, Guinea; 2 WHO country office, Guinea; 3 Ministry of Public health Cameroon

**Keywords:** Measles, immunization, evaluation, vaccination, coverage

## Abstract

**Introduction:**

To estimate the post-campaign level of measles vaccination coverage in Guinea.

**Methods:**

Interview of parents and observation of measles vaccination cards of children aged 9 to 59 months during the mass measles campaign. A nationwide cluster randomized sample under health District stratification.

**Results:**

64.2% (95%CI = 60.9% to 67.4%) of children were vaccinated and had their measles vaccination card. With respect to card and history 90.5% (95%CI = 88.3% to 92.3%) were vaccinated. The estimation was found to be between 72.7% and 81.9%. Coverage with card increased from 55.5% to 79.30% with the level of education of parents but that was not statistically significant, (X^2^(trend) =3.087 P= 0.07). However coverage with card significantly increased with profession from 55.1% for farmers followed by 59.2% for other manual workers to 73.8% for sellers, ending by 74.5% for settled technicians (X^2^ (trend) =12.16 P= 0.0005). For unvaccinated children, lack of information accounted for the main reason (37.03%) followed by parents’ occupation (23.45%), parents’ sickness (8.6%), children's sickness (4.9%) and others including vaccinators absent in the post or parents’ belief that it was a door to door campaign.

**Conclusion:**

The mass measles vaccination campaign achieved an approximate coverage of 75%. Although not enough for effective control of measles, it has covered an important gap left over by the routine immunization coverage 42%. Appropriate measures are needed to improve coverage in routine immunization and specific actions should be taken to target farmers and other manual workers’ families but also uneducated groups for both routine immunization and mass campaigns.

## Introduction

Measles still kills more than half of children who die annually from vaccine preventable-diseases. Among children surviving from measles, up to 10% will suffer of disabilities such as blindness, deafness and irreversible brain damage [1]. All six WHO regions have committed to measles elimination and five regions have set target dates. The America WHO Region achieved the goal in 2002, the western pacific Region aims at eliminating measles by the end of 2012 and the European and Eastern Mediterranean regions are accelerating their measles control activities in order to eliminate measles by 2015. In 2011 countries in the African region took on the goal to eliminate measles by 2020, and in 2010 the South-East Region adopted a resolution urging countries to mobilize resources to support the elimination of measles, the target date for which was under discussion [2]. In 2008 the world health assembly endorsed a target of 90% reduction in measles mortality by 2010 compared with 2000. Estimated global measles mortality decreased 74% from 535300 deaths in 2000 to 139300 in 2010 [3]. Accordingly, the World Health Assembly committed again in May 2010 to endorse a series of interim measles control targets for 2015 which include exceeding 90% coverage with first dose measles containing vaccine (MCV1) nationally, exceeding 80% vaccination coverage in every district, reducing annual measles incidence to < 5cases per million, maintaining that level and reducing measles mortality by more than 95% compare with 2000 estimates [4]. This commitment was set in line to achieve the Millennium development goals4(MDG4) which aim to reduce the overall number of death among children by two-thirds between 1990 and 2015 [5]. The proportion of children vaccinated against measles was adopted as an indicator to measure progress towards this MDG4 and the 2020 measles elimination objective in Africa region. The highly infectious nature of measles virus requires maintenance of very high levels of population immunity. Supplementary Immunization Activities(SIA) conducted every two, three or four years depending on the quality of routine immunization currently play an important role in protecting children in countries unable to achieve and maintain high and homogenous vaccination coverage through routine immunization systems [6]. In Guinea, the objective for the 2012 measles SIA was set at 95% vaccination coverage and the activities was implemented from June 29^th^ to July 5^th^ targeting estimated 2209623 children aged 9 to 59 months. Assessment was carried out 2 months later.

## Methods

A nationwide cross-sectional survey conducted 2months after measles national immunization days. Population was divided into homogeneous clusters. The list of clusters was provided by the national institute of demography. For each Health District one cluster was selected using a computer random process but two clusters for the two biggest Health Districts. Then a total of 40 clusters from the 38 Districts of the Country were randomly selected. 80 investigators were trained including role play to carry out data collection with a structured questionnaire. For each cluster, the investigators started in the middle and allocated numbers on folded papers to each of the four directions. Papers were tossed and the one selected gave the direction to follow. The first household on the direction was the starting one. In the household, when there were one or more target children, only one was selected using the same random process as for the selection of the direction to follow. The Child was observed and his immunization card was requested. A structured interview was then conducted to the parent in charge of him. At the end of the process investigators fully thanked the family and moved to the nearest household until 18 to 22 children were selected per cluster in order to have at least the expected sample of 840 children. In each household detailed explanation of the study objective was clarified to the family representative to have their informed consent. Data were recorded and analyzed using Epi-info software. Med-calc software was used to obtain the chi-square for trend and Health map software was used for mapping of coverage distribution per region.

## Results

**Figure 1:**
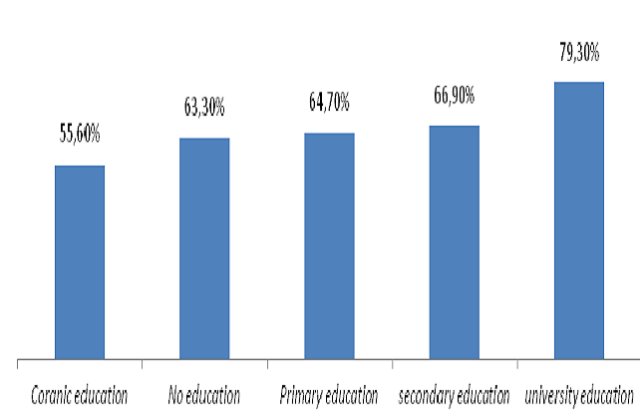
Vaccination coverage with card according to parents’ level of education

**Figure 2:**
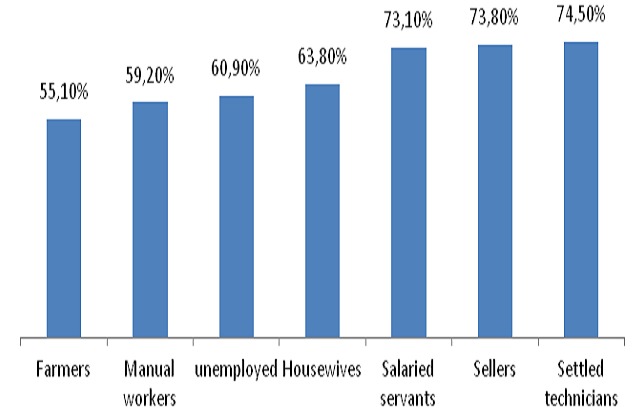
Vaccination coverage with card according to parents’ profession

**Figure 3:**
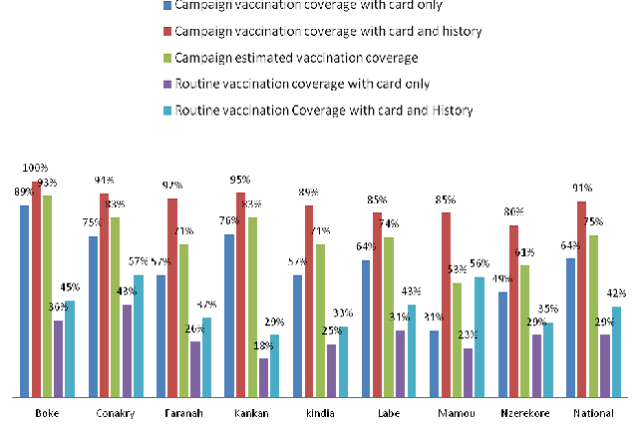
Comparison of routine and campaign measles vaccination coverage

866 households were investigated among which 853 (98.5%) with at least one target were selected for analysis. 549children were vaccinated and had their vaccination cards. The corresponding vaccination coverage was 64.2% (95%CI = 60.9% to 67.4%). These Children were aged 11 to 61 months and 50.4% were male. Mean age was 32.46 months with standard deviation of 14.03months. 226 children not having their vaccination card were reported as having a history of vaccination during the campaign from their parents’ declaration. Then a total of 775 children were vaccinated on the basis of vaccination cards and vaccination history during the campaign and the corresponding vaccination coverage was 90.5% (95%CI = 88.3% to 92.3%) Parents were aged 14 to 82 years and 71.7% were children's mothers. Single parent represented 12.8%. Mean age of parent was 31.53years with standard deviation of 11.35 Years. Parents were mostly uneducated, 55.2% and housewives, 51.8% (Table 1). 81 children, thus 9.5% (95%CI= 7.7% 11.7%) had no vaccination card and no history of vaccination during the campaign. Their mean was 27.48months with standard deviation of 12.13months. 48.1% were men. For reasons of non vaccination, lack of information accounted for 37.3%, parents’ occupation for 23.45%, parents’ sickness for 8.6%, children's sickness for 4.9%, vaccinators absent in the post during the visit for 3.7%, believes that the campaign was a door to door campaign for 3.7% and vaccination post far to reach for 2.46% (Table 2). Although there was an increase trend of coverage with vaccination card and the level of education, our data did not provide a strong relationship as the Chi square for trend was not significant (X^2^ trend = 3.08 P= 0.07’ > X^2^ trend = 3.08 P= 0.07) (Figure 1) However there was a significant increase trend of coverage and parents’ profession (X^2^(trend) =12.16 P= 0.0005’ > X^2^(trend) =12.16 P= 0.0005) (Figure 2). Vaccination Coverage varied from one region to another and we noted a constant overestimation of administrative coverage in all except Boke region (Figure 3).

**Table 1:** Characteristics of parents

Characteristics of parents		n	%
**Marital status**	Single Parents	109	12.8
	Married	743	87.2
**Relation with child**	Mothers	611	71.7
	Fathers	126	14.8
	Grand parents	58	6.8
	Brothers	57	6.6
**Education**	Others (Coranic etc...)	27	3.2
	No education	469	55.2
	Primary education	173	20.4
	Secondary education	151	17.8
	Tertiary education	29	3.4
**Profession**	farmers	107	12.7
	Others manual workers	49	5.8
	unemployed	46	5.5
	housewife	436	51.8
	Salaried servants	26	3.1
	sellers	122	14.5
	Settled technicians	55	6.5

**Table 2:** Reasons for non vaccinations

Reasons for non vaccination	n	%
Lack of information	30	37.03
parents absents or busied	19	23.45
Parents’ sickness	7	8.64
Children's sickness	4	4.93
Vaccinators absent in the post	3	3.70
Believes to a door to door campaign	3	3.70
Vaccination post far to reach	2	2.46
Ignorance of the importance	2	2.46
Age not out of range	1	1.23
Unknown	10	12.34

## Discussion

**Figure 4:**
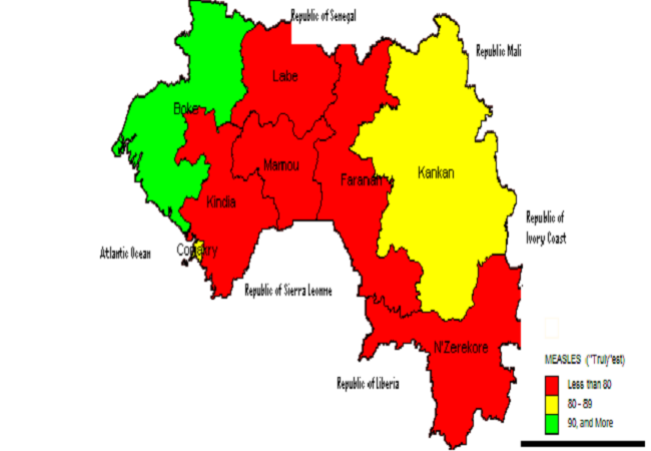
Map of estimated vaccination Coverage per region of Guinea

Let us admit that the “estimated vaccination coverage” (EVC) is the cut point to be used when talking about measles vaccination coverage in Guinea in 2012. The EVC lay between the proven vaccination coverage with card and the unsure vaccination coverage which encompassed children with vaccination cards and those with history of vaccination. Most often evaluation of measles campaigns are done immediately after to improve on recall bias. 2 months length time can be enough to consider the effect of a recall bias, especially when children have gotten additional routine vaccination during this length time confusion is likely to be possible. In fact children of unsure parents became randomly classified easily as vaccinated or not vaccinated depending on the subjectivity of the investigator of the household. Vaccination Coverage with history may overestimate if investigators are too sensitive or underestimate if they are too specific but these are unknown indicators. In assessing the validity of interview information in estimating community immunization levels in USA, Comstock observe among 494 people of all age that 99.2% were serologically immune whereas 83.4% were immune according to interview information. He further compared the validity of history per place of residence (rural or urban), annual income of head of households and age group and in all instances the proportion of immune persons was somewhat understated by interview results varying from 81.7% to 87.6% as compare to the 99.2%. However, although comparison for measles was inconclusive because nearly every participant was serologically immune, interview information was poorly correlated with the serological findings for mumps and poliomyelitis and understated for rubella and tetanus [7]. A study in hillsborough county,USA assessing correlation between history of either measles or vaccination and serologic immunity shown that positive history and positive antibody were concordant on 317 over 374 ( sensitivity of 84.8%) and negative history and negative antibody were concordant on 14 over 30(specificity of 47%) [8]. Applying these indicators to our unsure 226 children vaccinated based only on history, we can estimate that 226*0.85*0.47= 90 are effectively vaccinated and 136 are not effectively vaccinated, thus an “estimated coverage of (549 + 90)/853 = 75%”. A similar analysis was carried on in a comparable country in terms of anthropology, culture and socioeconomic status, Soudan. Variation rate between vaccination coverage with vaccination card and vaccination coverage with vaccination card plus history of vaccination was 23%. Our data showed a variation rate of 26%. The study further concluded that illiterate mothers had remarkably good recollections of their children measles vaccination status and therefore accurate estimation could rely only on mothers’ reports irrespective of the assumptions made about mothers who were unsure about children's vaccinations status. For example, if children of mothers who were unsure are assumed not to have received measles vaccine, the sensitivity of mother's reports would be 87% and the specificity 79% conversely, if such women are assumed to have vaccinated their children the sensitivity of mother's would then be 95% and the specificity would drop to 70% [9]. Applying these indicators to our unsure parents will provide the validity of the interview (Table 3). The “estimated vaccination coverage” based on the validity of the interview was between 72.7% and 81.94%, 75% is included in the interval and so can be a good approximation of the cut point for measles vaccination coverage in Guinea in 2012. Based on this cut point which takes into account the validity of the interview The distribution of coverage per region was estimated (Table 4) and a mapping generated (Figure 4).

**Table 3:** Validity of the interview results

	Children of unsure parents are assumed to haven't been vaccinated (Sensitivity 87% and specificity 79%)		Children of unsure parents are assumed to have been vaccinated (Sensitivity 95% and specificity 70%)	
**status**	Effectively vaccinated	Not effectively vaccinated	Effectively vaccinated	Not effectively vaccinated
Vaccinated with history (n = 226)	226-155 = 71	226*0.87*0.79= 155	226*0.95*0.70 = 150	226-150 = 76
Vaccinated with card (n = 549)	549	0	549	0
Effectively vaccinated	71 + 549= 620	155	150 + 549 = 699	76
Estimated vaccination coverage(EVC)	620/853*100= 72.7%	155/853*100 = 18.2%	699/853*100 = 81.94%	76/853*100 = 8.9%

**Table 4:** Distribution of coverage per region based on the validity of interview and the cut point

REGION	Number of children investigated	Number with proven vaccination card	Number with history of vaccination	Effectively vaccinated on history	Not effectively vaccinated on history	Estimated number of vaccinated	“Estimated vaccination Coverage” (%)
BOKE	109	97	12	5	7	102	93
CONAKRY	151	113	29	12	17	125	83
FARANAH	85	48	30	12	18	60	71
KANKAN	111	84	21	8	13	92	83
KINDIA	104	59	36	14	22	73	71
LABE	105	67	26	10	16	77	74
MAMOU	61	19	33	13	20	32	53
NZEREKORE	127	62	39	16	23	78	61
TOTAL	853	549	226	90	136	639	75

A catch up measles vaccination campaign is an opportunity to deliver booster and to catch up on children hard to reach due to poor socioeconomic conditions or geographical reasons in measles control country like Guinea. But when the trend of vaccination coverage increases with the socioeconomic level (profession and education) during mass vaccination as it is usually the case during routine immunization it suggests that efforts are still needed to improve on equity and the same who escaped routine immunization are probably left over. Missing to cover the gap due to socioeconomic discrepancy in the access to immunization is often observed during mass immunization [10] suggesting that specific actions are still needed to make mass immunization an equal opportunity for every child. However a coverage of 75% can be satisfactory if we consider measles routine coverage that is 42% [11] (Figure 3, unpublished data, Ministry of health, February 2012) and the experience of other countries in the past (9-75% in Burkina Faso, 10-95% in Nigeria, 40-75% in Columbia) [12] though not enough for effective control of measles. For example, the same experience was gotten in South Africa where a cluster survey conducted immediately before and two months after the mass campaign shown an increase from 55% for routine coverage to 72% for mass campaign coverage [12]. Measles campaigns have not always been satisfactory in low income countries and often haven't reached the estimated target [13, 14]. Despite extending the duration of the campaign in Kabul by 7 days and sending external monitors to search door-to-door for missed children, reported coverage was still low and a population-based cluster survey reported 86% coverage [15]. Lack of information has also been reported to be the main cause of non vaccination during mass immunization campaign in many other countries like Burkina Faso, Congo and South Africa. For the perspective of measles elimination in 2020, an important gap is still to be met for measles SIA campaign in Guinea in term of correlation between administrative coverage, 103% and the coverage of the survey post campaign, 75%. Experience of East European countries currently experiencing measles elimination proves that one good indicator can the good match between administrative coverage and that of the LQA coverage post campaign [16]. (Armenia, 96.8% &95.8%, Tajikistan 97.8% &96.6%, Turkmenistan 96% &97.6%) comparatively, an alternative for better handling of measles control and moving towards elimination would be: 1) Implementation of follow-up campaign given the very low capacity to provide second opportunity through routine immunization, 2) Introduction of LQA coverage surveys post-campaign. 3) Immunization efforts specifically targeting underserved groups (farmers and other manual workers, non classic educated and other non-educated).

## Conclusion

The mass measles vaccination campaign achieved an approximate coverage of 75%. Although not enough for effective control of measles, it has covered an important gap left over by the routine immunization coverage 42%. Appropriate measures are needed to improve coverage in routine immunization and specific actions should be taken to target farmers and other manual workers’ families but also uneducated groups for both routine immunization and mass campaigns.
